# Segmentation of the urothelium in optical coherence tomography images with dynamic contrast

**DOI:** 10.1117/1.JBO.26.8.086002

**Published:** 2021-08-14

**Authors:** Zhuo Xu, Hui Zhu, Hui Wang

**Affiliations:** a Miami University, Department of Chemical, Paper, and Biomedical Engineering, Oxford, Ohio, United States; b Urology Section Louis Stokes Cleveland Veterans Affairs Medical Center. Cleveland, Ohio, United States; c Cleveland Clinic Foundation, Glickman Urological and Kidney Institute, Department of Urology, Ohio, United States

**Keywords:** urothelium, optical coherence tomography, speckle, intracellular motion, dynamic contrast, bladder cancer

## Abstract

**Significance:** Speckle variation induced by intracellular motion (IM) in the urothelium was observed in optical coherence tomography (OCT) images. IM can be used as a dynamic contrast to segment the urothelium by comparing two sequential OCT images. This method opens the possibility of specifically tracking the distribution of urothelial cancerous cells for identifying the microinvasion of bladder tumors.

**Approach:** OCT images were acquired *ex vivo* with fresh porcine bladder tissue. IM was analyzed by tracking speckle variation using autocorrelation function, then quantified with constrained regularization method for inverting data (CONTIN method) to identify the decorrelation time (DT) of the speckle variations. Variance analysis was also conducted to show IM amplitude and distribution in the urothelium. The segmentation of the urothelium was demonstrated with OCT images with a visible urothelial layer and OCT images with an invisible urothelial layer.

**Results:** Significant speckle variation induced by IM was observed in the urothelium. However, the distribution of the IM is heterogeneous. The DTs are mostly concentrated between 1 and 30 ms. With the IM as a dynamic contrast, the urothelium can be accurately and exclusively segmented, even the urothelial layer is invisible in normal OCT images.

**Conclusions:** IM can be used as a dynamic contrast to exclusively track urothelial cell distribution. This contrast may provide a new mechanism for OCT to image the invasion depth and pattern of urothelial cancerous cells for accurately substaging of bladder cancer.

## 1 Introduction

Bladder cancer, the fourth most common cancer in men and the ninth most common in women in the USA, originates from the urothelium.^1^ Urothelial cancerous cells are initially confined in the urothelium, a tissue lining the lumen surface of the bladder, and may gradually invade the lamina propria (LP) and musculus propria (MP) underneath the urothelium. Therefore, tracking the invasion depth of urothelial cancerous cells is the cornerstone of stratifying patients into different stages for treatment planning. Previous clinical studies showed that optical coherence tomography (OCT) is a promising technology for bladder cancer diagnosis.^2^^–^^4^ In normal OCT images, normal bladder tissue with sufficient distention shows a clear boundary between the urothelium and the LP, due to different scattering coefficients. The boundary fades or disappears when urothelial cancerous cells invade the underlying LP. In clinics, the fading or disappearance of the boundary in normal OCT images is employed as the most important criterion to differentiate muscle-invasive and muscle-noninvasive bladder tumors. However, the invasion depth and the distribution patterns of the urothelial cancerous cells have been correlated with the prognosis of bladder cancer through pathohistological analysis but cannot be identified in normal OCT images for muscle-invasive bladder tumors.^5^^,^^6^ If OCT can specifically track urothelial cancerous cells instead of only relying on the boundary feature, physicians may employ the information to predict the recurrence and progression of bladder cancer and avoid over- and under-treatment.

In the last decade, aquaporins (AQPs), or water channels, have been identified on urothelial cells.^7^^–^^10^ Although the exact function is still not clear, these AQPs seem related to water transport. With fresh porcine tissue samples, we have directly visualized the dynamic process of water absorption and extraction through the urothelium. After absorbing water, we also observed significant intracellular motion (IM) in the urothelium. IM originates from the motion of molecules and organelles in the cytoplasm of eukaryotic cells and is essential for the proper functioning of cells.^11^ The technology of tracking IM can be divided into two categories. Fluorescence labeling methods, such as fluorescence photobleaching recovery kinetics,^12^ fluorescence correlation spectroscopy,^13^ and time-resolved fluorescence imaging,^14^ use fluorescence to quantify IM by tracking specific molecules. In contrast, coherent gated methods measure speckle variation induced by IM. Initially, holographic optical coherence imaging was used to image the IM of tumor spheroids and drug responses.^15^^,^^16^ Later, with OCT, IM has been used as an endogenous contrast to reveal the cellular and subcellular structures of freshly excised tissue. These cellular and subcellular structures are hardly visible in normal OCT images but have different mobilities, which can be extracted through standard deviation or power frequency analysis.^17^^,^^18^ Autocorrelation function analysis and the constrained regularization method for inverting data (CONTIN method) also have been adapted to quantify IM.^19^^,^^20^ The uniqueness of the coherence gated method is that it can detect IM at different depths without requiring fluorescence tagging. Therefore, the imaged objects can retain at a more natural status.

Here, we show that IM can be used as dynamic contrast to segment the urothelium. In this study, we characterize the IM in the urothelium through autocorrelation analysis of speckle variation and quantifies speckle decorrelation time (DT) with the CONTIN method. Due to the short speckle DT, it is feasible to use IM as dynamic contrast to specifically segment the urothelium with only two sequential OCT images, even when the boundary between the urothelium and the LP is invisible in normal OCT images.

## 2 Materials and Methods

### 2.1 Optical Coherence Tomography

The OCT used in the studies is based on spectral-domain OCT and was designed and built in-house. A superluminescent diode (SLD, Inphenix) centered at 840 nm with a full width at half maximum bandwidth of 45 nm was used as the broadband light source. The spectrometer in the system consists of a transmission grating at 1200  l/mm at 840 nm (Wasatch) and a 2048-pixel linear CCD (AVIIVA SM2). The measured axial resolution is ∼7.5  μm, and the calculated lateral resolution is ∼7.9  μm based on the collimated lens (AC127-019, Thorlabs), the focusing lens (AC250-030, Thorlabs), and the fiber mode field diameter (750HP). The system can acquire images at 20K one-dimensional scans (A-scans) per second.

### 2.2 Porcine Bladder Tissue and Histology

The porcine bladder tissue was obtained from a local slaughterhouse immediately after the animals were euthanized. The tissue was kept in 4°C Krebs–Henseleit solution before use. The bladder was first flushed with DI water and then cut into smaller pieces (∼5  cm×5  cm). During imaging, a tissue sample was clipped from the four edges and mounted on four translational stages. By moving the translation stages in four directions with the same distance, the tissue sample can be uniformly stretched. A pin was used to mark the position of the imaged region during OCT imaging. A 5×5  mm tissue block was extracted from the marked location and processed according to standard histological sample preparation. Hematoxylin and eosin (H&E) were used to stain several 7-μm-thick sections taken from the middle of the tissue block.

### 2.3 Autocorrelation Function Curve

For autocorrelation analysis, we positioned the light spot at the same location on a tissue sample and acquired an image (M-scan) with 80K A-scans at 20K A-scans/s. The background from the sample arm was removed by subtracting a prerecorded image when the light in the sample arm was blocked.

The 80K A-scans were divided into 20 segments. The autocorrelation function curve (AFC) at a specific depth z along an A-scan of each segment, G(τ,z), was calculated as

$$G(\tau ,z)=\frac{\left\langle I(z,t)I(z,t+\tau \right\rangle }{{\left\langle I(z,t)\right\rangle }^{2}},$$ (1)

where ⟨I(z,t)⟩ is the intensity by calculating the squared norm of the OCT signal at z, and τ is the time delay of the autocorrelation. To reduce the variation, the AFC used for analysis is the mean of the AFCs of the 20 segments.

### 2.4 CONTIN Method

In this study, the CONTIN method was employed to estimate the DT of the speckle variation in the urothelium. The CONTIN method has been commonly used in dynamic light scattering (DLS) to estimate the diameters of the particles dispersed in a solution. The AFC of the light intensity variation induced by the random motion of particles can be fitted by multiple exponential decay functions. Each exponential decay function represents a particle-related diffusion rate in the solution. A particle moving fast corresponds to an exponential decay function with a short decay time. In OCT, the AFC of the light intensity variation at different depths can be calculated using an M-scan (Sec. 2.3). As the light intensity variation in OCT can be viewed as speckle variation shown in OCT images, the decay time represents speckle DT. Assuming random motion of cell organelles in urothelial cells, we can fit the AFCs with multiple exponential decay functions as

$$G_{m}(\tau ,z)=A_{m,n}\cdot x_{n},$$ (2)

where $G_{m}(\tau ,z)$ is a vector of the AFC data at a depth z and $x_{n}$ is the contribution of exponential decay functions with different DTs, $\gamma_{n}$. $A_{m,n}$ is a transfer matrix connecting $G_{m}(\tau ,z)$ and $x_{n}$, and defined by $A_{m,n}=\exp(-t_{m}/\gamma_{n})$. Solving $x_{n}$ from Eq. (2) is an ill-posed problem. The CONTIN method addresses this issue by including an additional constraint as a regulation term to seek the minimum value of D(α) as

$$D(\alpha )=\|Ax-G\|+\alpha^{2}{\left\|\Omega x\right\|}^{2}.$$ (3)

Here, the first term is the residual norm, and the second term represents the cost of the additional constraint, and α is the regularizer. An L-curve can be plotted as the residual norm versus the additional cost at different values of α. The α at the corner represents a balance between these two errors and is considered as the optimal value for the best fitting.^21^ In this study, the additional constraint, Ω, assumes that the distribution of the speckle DTs of the cellular organelles was continuous and smooth. The AFC and the optimal α were fed into the CONTIN method to calculate the contribution of different decayed exponential functions.

### 2.5 Variance Analysis

A speckle variance image was calculated from 20 continually recorded frames by scanning the same location of a tissue sample. The images were acquired at 20  frames/s with 1000 A-scans per frame across 0.5 mm. The variance of each pixel over 20 frames was calculated and then normalized to produce a color-mapped image to represent the variance of each pixel.

### 2.6 Segmentation of the Urothelium

For segmentation, we picked two sequential OCT images acquired at 20  frames/s. We adapted the algorithm used in optical microangiography to segment the urothelium based on the speckle variation.^22^ After removing the DC background, the difference of the i’th A-scan between the two frames, $D_{i}$, was calculated as

$$D_{i}=\frac{\mathrm{FT}(sf_{1i}-sf_{2i})}{abs[\mathrm{FT}(sf_{1i}+sf_{2i})]/2},$$ (4)

where $sf_{1i}$ and $sf_{2i}$ are the corresponding spectral fringe of an A-scan, and FT is Fourier transform. The difference was normalized to the averaged OCT signal amplitude of the two frames.

## 3 Results

### 3.1 Speckle Variation in the Urothelium

**Fig. 1:**
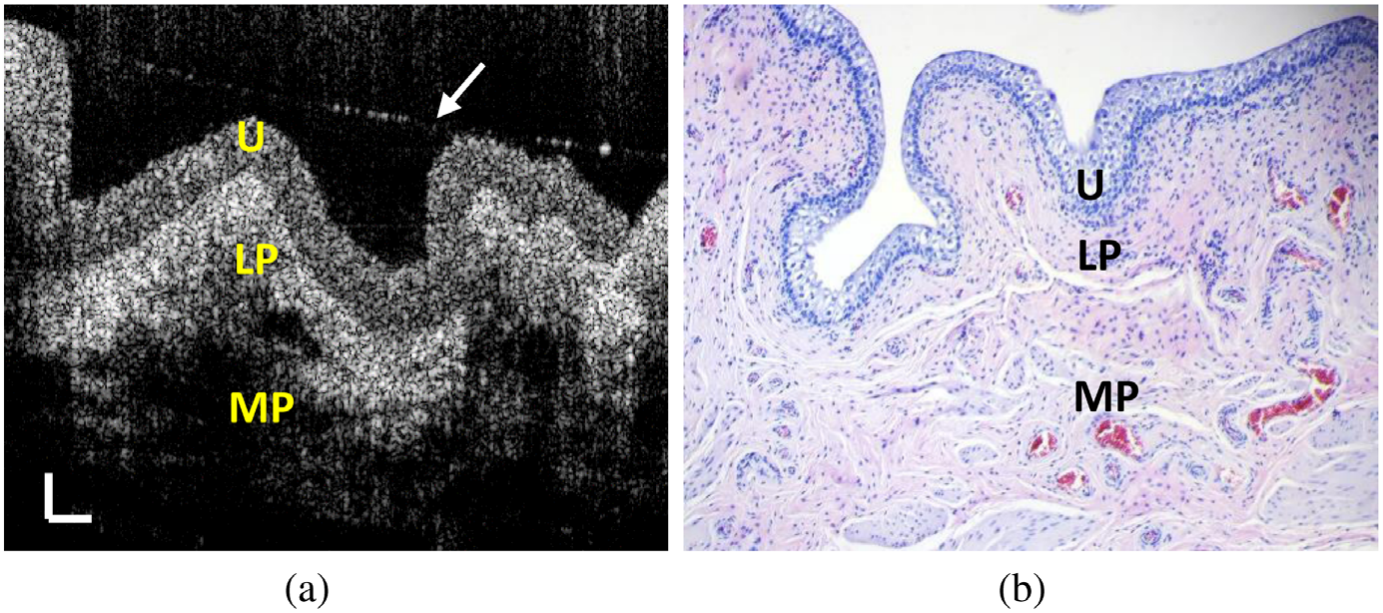
OCT images of a fresh porcine bladder tissue sample under a stretched condition. (a) A normal OCT image, (b) corresponding H&E histological image (20×), and (c) 100 OCT images captured at 20  frames/s. The arrow in (a) marked the interface of a thin water layer over the tissue surface. U, urothelium; LP, lamina propria (LP); MP, muscularis propria (scale bar: 0.1 mm) (Video 1, MP4, 2 MB [URL: https://doi.org/10.1117/1.JBO.26.8.086002.1]).

Normal OCT images were acquired on a stretched bladder tissue sample covered with a thin water layer, as shown in Fig. 1(a). Figure 1(b) shows the corresponding histological image of the imaged tissue. During imaging, the area of the tissue sample was stretched by four times. Under OCT, the boundary between the urothelium and the LP is clearly visible. The layered structure shown in the OCT image correlates well with the histological image in Fig. 1(b). In Video 1, we have continually acquired 100 OCT images at 20  frames/s. Significant IM, manifested as speckle variation, can be observed in the urothelium.

### 3.2 Autocorrelation Analysis

**Fig. 2:**
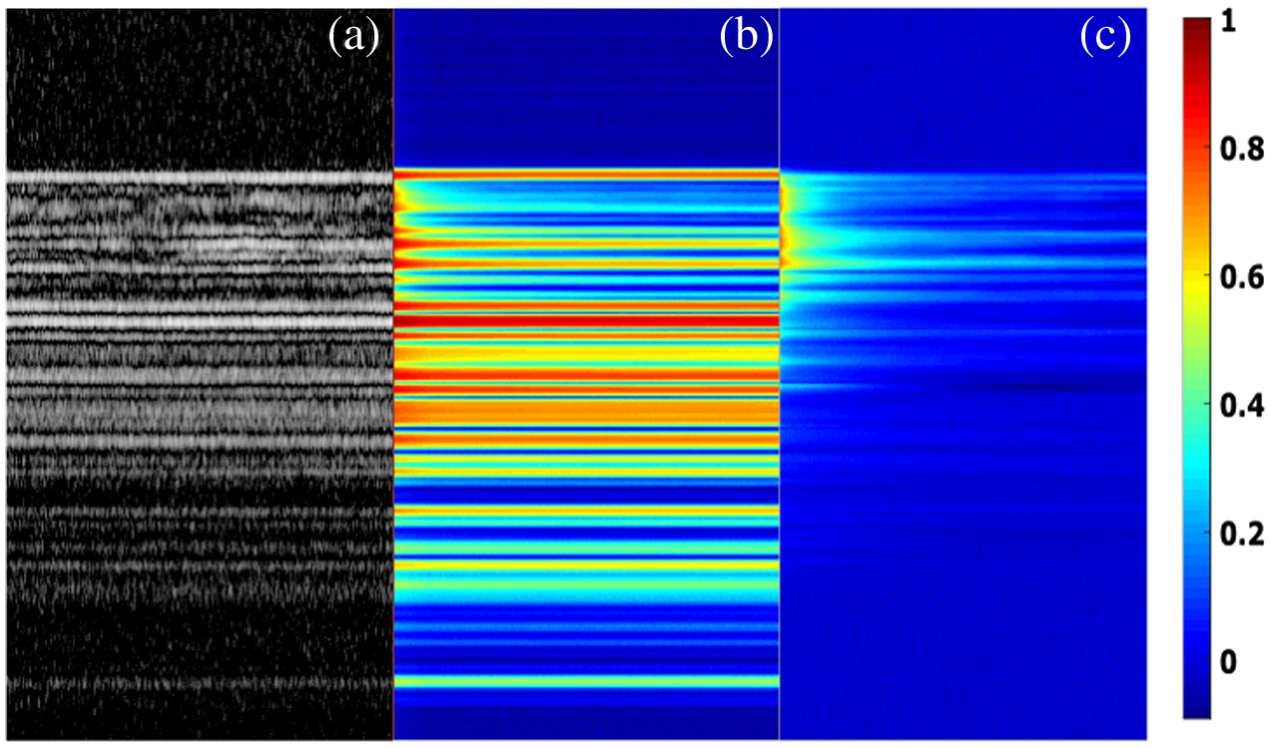
Autocorrelation color maps. (a) An OCT M-scan acquired from a porcine bladder tissue sample. (b) The autocorrelation color map calculated from the OCT M-scan shown in (a), (c) The autocorrelation color map after removing the mean value of (a).

From the intensity variation in an M-scan, we calculated the AFCs at different depths. Figure 2(a) shows an M-scan image composed of 500 A-scans. We can observe different degrees of intensity variation at the different depths of the M-scan due to the IM in the tissue. The autocorrelation color map, shown in Fig. 2(b), was the average of the AFCs of the 20 segments described in Sec. 2.3. The static tissue shows constant AFCs, whereas the tissue with IM has decaying AFCs. Figure 2(c) shows the AFCs calculated from the M-scan after subtracting the mean intensity value of each pixel and normalized to the autocorrelation value at time delay τ=0. By removing the mean intensity, only the dynamic part of the M-scan remains. Gradually decaying ACFs with long tails concentrated in the top layer, the location of the urothelium. For the static tissue, because the mean intensity values have been removed, the speckle intensity variations mostly came from the random background noise, whose AFCs were shown as a sharp peak at τ=0 without having significant tails.

**Fig. 3:**
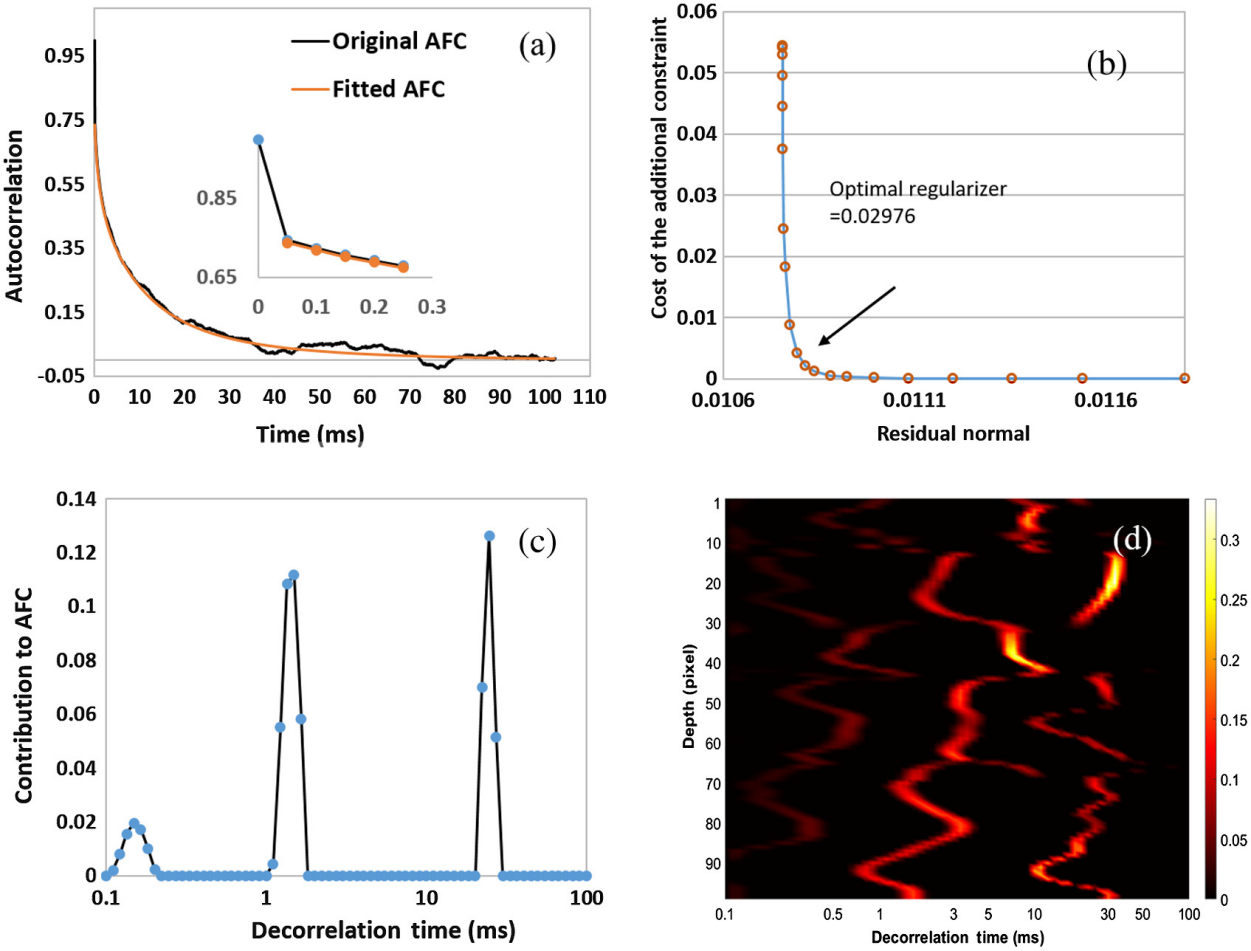
Autocorrelation analysis with the CONTIN method. (a) A typical AFC with IM and the recovered AFC with the DT peaks identified in (c) with the CONTIN method. (b) L curve employed to find the optimal regularizer from the AFC. (c) The DT derived from the AFC shown in (a) with the CONTIN method. (d) DT color map of 100 pixels with IM calculated with CONTIN method. The inset in (a) is the magnified AFC at time delay τ=0.

We employed the CONTIN method to analyze the ACFs with significant IM shown in Fig. 2(c). Figure 3(a) shows a representative AFC calculated from the speckle intensity variation at a fixed depth. The AFC has two segments. The first segment has a quick drop from 1 to ∼0.75 in less than 0.05 s, which is shown as the magnified inset in Fig. 3(a). The second segment following the first segment has a gradually decaying tail lasting ∼50  ms. The CONTIN method can identify three peaks at 0.165, 1.49, and 24.6 ms, as shown in Fig. 3(c). The three peaks represent three major speckle DTs, the reciprocals of the decay rates of the exponential functions. To confirm that the DT peaks have been identified correctly, we recovered the second segment of the AFC with the exponential decay functions having the DTs in Fig. 3(c). The AFC and the recovered AFC are matched very well, as shown in Fig. 3(a). In Fig. 3(d), we also show the DT distribution of 100 continuous pixels with significant IM as a color map. Clearly, the IM is distributed heterogeneously, but significant DT peaks are in a range between 1 and 30 ms.

### 3.3 Variance Analysis

**Fig. 4:**
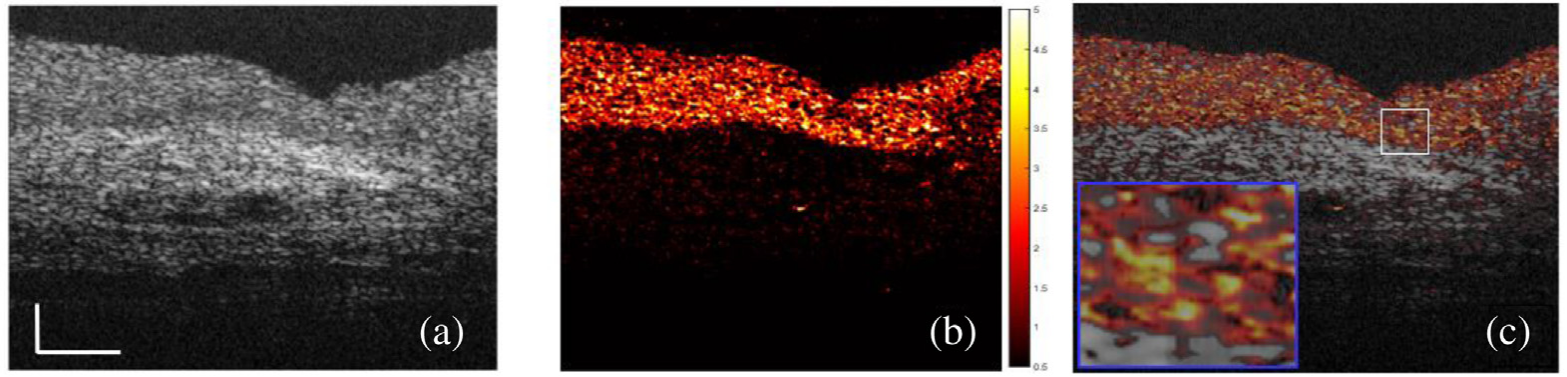
Speckle variance analysis. (a) An OCT image after averaging 20 continuously acquired OCT images at 20  frames/s. (b) Normalized speckle variance image of the 20 OCT images; the color map indicates the amplitude of the variance. (c) The fused image of (a) and (b) with an enlarged view (blue box) of the white box (scale bar: 0.1 mm).

Variance analysis was conducted to show the amplitude and the distribution of IM. Figure 4(a) shows an averaged image from 20 sequentially acquired OCT images. The variance of each pixel was calculated and shown in Fig. 4(b). Figure 4(c) shows the fused image of Figs. 4(a) and 4(b). As the boundary between the urothelium and the LP is visible in Fig. 4(a), we can find that the distribution of intense IM matches the urothelium very well, as shown in Fig. 4(c). However, the IM does not distribute uniformly in the urothelium. By looking closely at the magnified view shown in Fig. 4(c), some regions of the urothelium show intense IM, whereas the other regions only show minor IM similar to those in the LP. Due to the limitation of our OCT system’s resolution, we cannot visualize the cellular and subcellular structures and tell which organelles introduce significant IM.

### 3.4 Segmentation of the Urothelium with Intracellular Motion

**Fig. 5:**
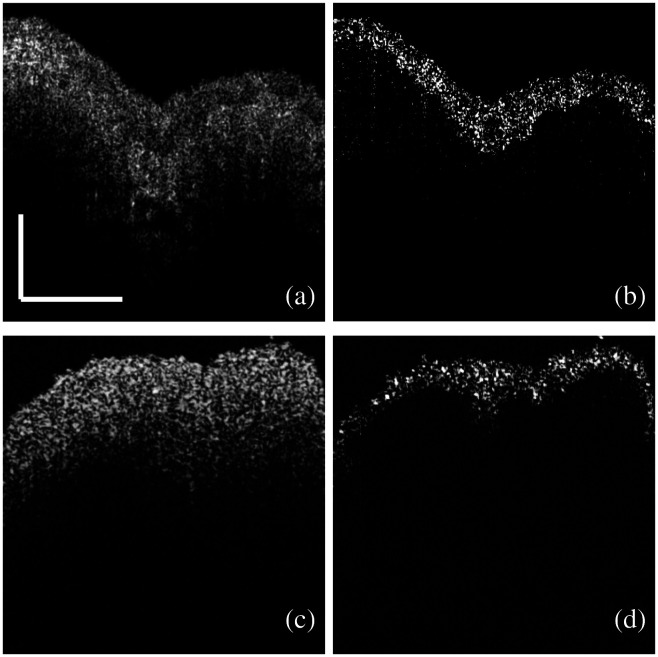
Segmentation of the urothelium with IM. (a) An OCT image of a fresh porcine bladder tissue sample with significant stretching. (b) The image reconstructed after the subtraction of the OCT complex signals between the two sequential images like (a). (c) An OCT image of a fresh porcine bladder tissue sample with minimal stretching. (d) The image reconstructed after the subtraction of the OCT complex signals between the two sequential images like (c) (scale bar: 0.5 mm).

In Fig. 4, we have observed that the variance of the IM overlapped with the urothelium very well. In Fig. 5, we demonstrated the feasibility of using IM as a dynamic contrast to segment the urothelium with only two sequentially acquired images. Figure 5(a) shows a normal OCT image of a significantly stretched bladder tissue sample. The boundary between the urothelium and the LP is clear. Figure 5(b) shows the segmented urothelium using the complex difference of two sequential OCT images, described in Sec. 2.6. The tissue with significant IM closely matches the shape and the boundary of the urothelium, which is visible in Fig. 5(a). This result gives us the confidence to use IM as dynamic contrast to exclusively extract the urothelium. Figure 5(c) shows a normal OCT image of a bladder tissue sample with minimal stretching. We cannot observe the boundary between the urothelium and the LP in the normal OCT image due to the minimal stretch [Fig. 5(c)]. However, using the IM as the contrast, the urothelium can be extracted exclusively, as shown in Fig. 5(d).

## 4 Discussion

The urothelium consists of three types of cells: the superficial umbrella cells, the intermediate cells, and the basal cells interfacing with the LP.^23^ Conventionally, the urothelium is considered a passive barrier between the blood and the urine. Transmission electron microscopy reveals that the apical surface of the umbrella cell is covered with plaques, which are interconnected by hinges.^24^ Although the function of this plaque–hinge structure is still not clear, they may contribute to the barrier function of the urothelium. Adjacent umbrella cells are tightly bound by tight junctions, which seal the lateral intercellular space to prevent paracellular flux across the urothelium. However, AQPs have been discovered in the human, rat, and porcine urothelium, challenging the exclusive barrier function of the urothelium and suggesting that water can be transported into the urothelium.^7^^–^^10^ Numerous organelles are distributed in urothelial cells, such as mitochondria, endoplasmic reticulum, nucleus, glycogen granules, lamellar body filled with lipid droplets, and unique fusiform-shaped vesicles.^23^^,^^25^ Due to the limited resolution of the OCT, we cannot directly observe these organelles in images. However, the backscattered photons from the organelles within the coherent volume of an OCT beam can form speckles shown as irregular patterns in OCT images. Such speckles are very sensitive to the organelles’ motion up to the nanometer scale.^26^ Therefore, the organelles’ motion is reflected as the intensity variation of the speckles in OCT images. As long as the surface of the urothelium is covered with a thin layer of water, we can observe significant IM in the urothelium, as shown in Video 1.

We conducted quantitative analyses of the IM in the urothelium using two methods based on the speckle variation. The organelles’ diffusion rates are varied at different depths of the urothelium. A slow diffusion or speckle variation has a gradually reduced AFC with a long tail, whereas static tissue has a single-peak AFC at τ=0 when the mean values were removed, as shown in Fig. 2. With the CONTIN method, we can fit the AFCs with exponential decay functions, which are characterized as speckle DTs. DT is the duration for dynamical speckles to become uncorrelated. According to DLS,^20^ the reciprocal of a DT is proportional to a diffusion rate. We calculated the DT distribution of 100 continuous pixels in the urothelium. The IM at different depths has three or four DT peaks with major contributions from the peaks between 1 and 30 ms. The multiple DT peaks suggest that several different diffusion patterns exist simultaneously in the coherent volume of OCT. This observation can be attributed to the different sizes, shapes, and densities of the organelles in the urothelium. It also implies that as long as two OCT images are acquired with a time interval longer than 30 ms, we should observe apparent speckle variations. The AFC analysis cannot directly tell us the motion amplitude of the IM in the urothelium, so we conducted variance analysis. We observed that the tissue with intense IM overlapped with the urothelium very well, and the distribution of IM is heterogeneous. The dynamical regions with large variances are mixed with the static regions with minimal variances. This observation indicates that not everywhere in the urothelium has intense IM. In addition, intense IM seems mostly located around the middle and the lower part of the urothelium. The top layer of the urothelium is formed by the umbrella cells, which are large polyhedral cells with a size up to 250  μm after stretching.^24^ The umbrella cells have multiple large nucleates (∼5 to 10  μm) and are filled with fusiform-shaped vesicles and other organelles. These organelles are mostly located below the central part of the umbrella cells.^25^ The lack of dense organelles may explain the lower IM contrast in the sub-apical regions of the urothelium.

When imaging normal bladder tissue with significant stretching under OCT, we can observe a clear boundary between the urothelium and the LP as shown in Fig. 1. The urothelium can be stretched easily to accommodate the volume change of the bladder. When stretched, the urothelial cell tends to become longer, thinner, and flatter. These changes result in a sparser distribution of the organelles in the cells along the bladder surface compared with that of the non-stretched bladder. Therefore, the light scattering from the urothelium is significantly reduced after stretching. In contrast, the LP consists of several types of cells, such as fibroblasts, adipocytes, and nerve endings, surrounded by the extracellular matrix (ECM) full of elastic fibers.^27^ When the LP is stretched, the space of the ECM between the cells in the LP tends to be compressed, so the density of the cells in the ECM is increased, leading to enhanced light scattering. Therefore, in a normal OCT image, a clear boundary can be observed between the urothelium and the LP when the tissue is stretched sufficiently, due to different scattering strengths. Histologically, this boundary is the location of the basement membrane,^28^ a single layer separating the urothelium and the LP. In clinics, layered structure in the normal bladder tissue observed under OCT can be attributed to the significantly stretched bladder after filling with saline. However, such a boundary cannot be clearly visualized when a bladder tissue is not stretched or stretched minimally, as shown in Fig. 5(c).

Bladder cancer, including papillary tumors and carcinoma *in situ* (CIS), originates from the urothelium.^29^ OCT has shown the potential to differentiate muscle-noninvasive and muscle-invasive bladder cancer based on the visibility of the boundary when the bladder is stretched sufficiently.^2^^,^^3^ Usually, the fading or disappearance of the boundary in an OCT image indicates that the urothelial cancerous cells have invaded the LP or the MP. For papillary tumors, OCT images have to be acquired from the stalk base of a papillary tumor instead of the top of the papillary surface because stretching the bladder cannot create even tension over the papillary top surface for clearly visualizing the boundary.^30^ However, manipulating an OCT fiber probe through a cystoscope to image the base of a papillary tumor is inconvenient. In previous small cohort and single-center clinical studies, the sensitivity and specificity of diagnosis by combing OCT with white-light cystoscopy were ranged between 75% and 100% and 65% and 89%.^30^ Based on a recent multi-center phase II trial, the sensitivity of staging papillary bladder tumors is 64.7%.^31^ All these studies used clinical pathohistological diagnosis as the reference. We should be aware that the accuracy of staging T1 tumors, whose cancerous cells have spread to the LP, using clinical pathohistological diagnosis is only ∼50%.^32^^,^^33^ These facts suggest that the visibility of the boundary may not be a reliable diagnostic criterion for staging bladder tumors. Recently, the invasion pattern and depth of the urothelial cancer cells from the basement membrane, termed as microinvasion, has been correlated with the prognosis of bladder cancer.^5^^,^^6^ It is clinically significant if OCT can specifically track urothelial cancerous cell distribution, whether the boundary is visible or not.

We have shown that we can segment the urothelium specifically by calculating each pixel’s variance with 20 sequential images, as shown in Fig. 4 while this method is not realistic in clinics due to the long image acquisition time. Using only two sequential images acquired at 20  frames/s, we showed that the urothelium could be specifically segmented using the IM as dynamic contrast. The contrast comes from the phase and intensity differences of the dynamic speckles between the two sequential images. We simulated a muscle-invasive bladder tumor using a minimally stretched bladder tissue sample, as the boundary was not visible for the minimally stretched bladder tissue sample shown in Fig. 5(c). The urothelium can be extracted due to the IM in the urothelium even when the boundary is not visible in the normal OCT image, as shown in Fig. 5(d).

Our study has shown that active IM exists in the urothelium. The speckle variations induced by the IM can be used as dynamical contrast to specifically segment the urothelium even when the boundary is not visible. This capability might open a new route to *in vivo* detection of microinvasion in papillary bladder tumors and CIS, which is a flat but highly risky tumor.^34^^,^^35^ Detection CIS is challenging under white light cystoscopy. Under OCT, CIS shows blur and discontinued basement membrane due to cytologic changes of the urothelial cancerous cells but often confounded with inflammatory tissue.^28^^,^^35^ Specifically tracking of the urothelial cells with dynamic contrast might allow us to visualize the distribution of cancerous urothelial cells in CIS, leading to better detection of CIS.

Due to the limited resolution and contrast, we cannot identify the organelles that contribute to the IM. Combining fluorescence imaging with OCT may help us to answer this question in the future. Our imaging interval between two sequential images was ∼50  ms, which is longer than the maximum DT, ∼30  ms. Therefore, it is possible to acquire images at 30  frames/s, which will help to reduce human body bulky motion artifacts. All images in this study were *ex vivo* obtained with fresh porcine tissue as done by other dynamical imaging studies.^16^^–^^18^ It should be recognized that the IM may be dominated by Brownian motion for excised tissue. For *in vivo* studies, super diffusion (active transport), such as motor-driven organelles along microtubules, may play a vital role.^36^^,^^37^ The more intense *in vivo* IM may allow us to employ OCT at an even higher imaging speed. Therefore, it is critical to verify the results through *in vivo* studies in the future. In previous clinical studies,^2^^–^^4^ forward-looking fiber catheters were used in conjunction with a rigid cystoscope to acquire images in less than 1  frame/s. To our knowledge, high-speed Fourier-domain OCT with a forward-look fiber catheter has not been tested in urological clinics, but it is technically feasible to adapt the system used for other clinical applications.^38^

In conclusion, we have observed intense IM existing in the urothelium. Quantitative analyses showed that the IM was heterogeneously distributed in the urothelium with major speckle DTs peaked between 1 and 30 ms. Such intense IM can be used as dynamical contrast to segment the urothelium with only two sequential OCT images. We believe that this new contrast can be used for *in vivo* tracking the invasion of the urothelial cancerous cells to the LP and the MP, but it needs to be verified through future clinical studies.
